# A Critical Assessment of the Therapeutic Potential of Resveratrol Supplements for Treating Mitochondrial Disorders

**DOI:** 10.3390/nu9091017

**Published:** 2017-09-14

**Authors:** Boel De Paepe, Rudy Van Coster

**Affiliations:** 1 Neuromuscular Reference Centre, Ghent University Hospital, 9000 Ghent, Belgium; 2 Department of Pediatrics-Division of Pediatric Neurology and Metabolism, Ghent University Hospital, 9000 Ghent, Belgium; rudy.vancoster@ugent.be

**Keywords:** mitochondrial biogenesis, mitochondrial disorders, oxidative phosphorylation, resveratrol

## Abstract

In human cells, mitochondria provide the largest part of cellular energy in the form of adenosine triphosphate generated by the process of oxidative phosphorylation (OXPHOS). Impaired OXPHOS activity leads to a heterogeneous group of inherited diseases for which therapeutic options today remain very limited. Potential innovative strategies aim to ameliorate mitochondrial function by increasing the total mitochondrial load of tissues and/or to scavenge the excess of reactive oxygen species generated by OXPHOS malfunctioning. In this respect, resveratrol, a compound that conveniently combines mitogenetic with antioxidant activities and, as a bonus, possesses anti-apoptotic properties, has come forward as a promising nutraceutical. We review the scientific evidence gathered so far through experiments in both in vitro and in vivo systems, evaluating the therapeutic effect that resveratrol is expected to generate in mitochondrial patients. The obtained results are encouraging, but clearly show that achieving normalization of OXPHOS function with this strategy alone could prove to be an unattainable goal.

## 1. Introduction

Mitochondria are the powerhouses of human cells and generate the bulk of energy necessary for cell maintenance and growth in the form of adenosine triphosphate (ATP), through the process of oxidative phosphorylation (OXPHOS). The OXPHOS system consists of five multiprotein complexes that associate with the inner mitochondrial membrane: complex I (NADH: ubiquinone oxidoreductase), complex II (succinate: ubiquinone oxidoreductase), complex III (ubiquinol: cytochrome c oxidoreductase), complex IV (cytochrome c oxidase), and complex V (ATP synthase). Reducing equivalents are delivered via NADH, ubiquinol, and cytochrome c, allowing high energy electrons to pass along the first four complexes, a process that releases the energy needed to pump hydrogen into the inter-membrane space, converting O_2_ to H_2_O. The proton gradient that develops between the mitochondrial matrix and the inter-membrane space generates an electrochemical membrane potential that drives the conversion of ADP to ATP by complex V. Both chromosomal and mitochondrial DNA (mtDNA)-encoded genes are essential for OXPHOS functioning. The mitochondrial genome is polyploid with multiple copies of mtDNA present within each individual mitochondrion and can exhibit heteroplasmy, i.e., the co-existence of different genetic variants within the same cell. The mtDNA encodes for 13 structural subunits of complexes I, III, IV, and V and for 2 rRNAs and 22 tRNAs necessary for mitochondrial protein translation. All other components of the OXPHOS machinery are encoded by the nuclear genome, and the latter controls all other aspects of mitochondrial functioning and nuclear–mitochondrial communication [1]. There are more than 70 nuclear genes encoding structural OXPHOS components. A myriad of other nuclear gene products are involved in regulation, post-translational modification, signaling, importation, folding, and assembly of the OXPHOS components. All these factors are equally essential for proper OXPHOS functioning [2].

Mitochondrial OXPHOS defects are estimated to affect approximately 1 in 5000 children born, and are associated with a broad spectrum of clinical manifestations ranging from mild myopathy to lethal multi-system disorders [3]. Isolated complex I deficiency [4] and mtDNA depletion syndrome [5] are the most frequent underlying molecular causes. Due to the dual origin of the genes involved, and the specific nature of the mitochondrial genome, OXPHOS defects are complex. In case of heteroplasmic mtDNA mutations, disease develops only if the mutant-load of a tissue reaches the threshold required to reduce OXPHOS capacities. The amount of mutant mtDNA determines severity of symptoms ranging from mild disease to debilitating and lethal conditions. Point mutations are mostly heteroplasmic, displaying considerable clinical heterogeneity. They may occur within protein, tRNA, or rRNA genes, but more than half of disease-related point mutations reported are located within mt-tRNA genes. Over 80% of patients with mitochondrial encephalomyopathy, lactic acidosis and stroke-like episodes (MELAS) have the m.3243A>G mutation in the MT-TL1 gene, and myoclonic epilepsy and ragged red fibers (MERRF) is caused most commonly by an m.8344A>G point mutation in the MT-TK gene. Neuropathy, ataxia, and retinitis pigmentosa (NARP) is usually due to the MT-ATP6 m.8993T>G mutation [6].

While we await the coming of age of gene therapies for human monogenic diseases, the development of effective pharmacological therapies for OXPHOS deficiencies has been extremely limited so far [7]. Nutritional supplementation has been tried: coenzyme Q10, vitamin C, creatine, sodium dichloroacetate, sodium pyruvate, and L-arginine. This generated some evidence of improved symptoms or slower progression in subsets of patients, but a pressing need for frontline treatments remains. A possible therapeutic approach for OXPHOS deficiencies is to increase the number of mitochondria per cell, which can result in greater capacities to produce ATP. Another strategy is to reduce the cell-damaging side effects of dysfunctional OXPHOS, i.e., the generation of harmful reactive oxygen species (ROS) and cell death. Along this line, resveratrol (RSV) seems an exquisite candidate, as the compound has been observed to possess mitogenetic, antioxidant, and anti-apoptotic activities.

## 2. The Stimulatory Effect of Resveratrol on Mitogenesis and Mitochondrial Interconnections

Mitochondrial biogenesis is a highly synchronized process driven by changing energetic demands. Regulation is governed by a complex system of transcription factors and co-activators, in which the peroxisome proliferator-activated receptor-γ coactivator α (PGC-1α) functions as a master regulator [8,9]. Stimulation of PGC-1α activity results in increased mitochondrial mass and function. PGC-1α expression in its turn is controlled by the peroxisome proliferation activated receptor family (PPAR) [10] and by deacetylation mediated by NAD+-dependent protein deacetylase sirtuin-1 (SIRT-1) [11,12]. In addition to sufficient amounts of mitochondria to achieve desirable ATP levels in the cell, interconnection of mitochondria into a complex cell-spanning network is another requisite for proper OXPHOS functioning. The mitochondrial network takes shape through the dynamic equilibrium between fusion and fission of the organelles, complexly regulated by changing cellular conditions [13] through the activities of mitofusins (MFN) and dynamins. Mitochondrial interconnection promotes the sharing of constituents and positively influences overall efficiency of OXPHOS [14]. Fragmented mitochondria are known to display diminished respiratory capacity [15].

Higher mtDNA content and increased mitogenesis have been suggested to improve the disease phenotype of patients with OXPHOS deficiencies. Mitochondrial proliferation was shown to protect mutation carriers from developing Leber’s hereditary optic neuropathy [16]. Heteroplasmic OXPHOS defects benefit from intensive exercise, an effect attributed to PGC-1α-induced mitochondrial stimulation [17]. In cybrids harboring the classical m.3243A>G MELAS mutation, PGC-1α overexpression improved OXPHOS complex III and complex IV deficiency [18]. Evidence accumulates of a positive effect of RSV on the process of mitogenesis mediated via PGC-1α stimulation. By inhibiting phosphodiesterases, RSV has been observed to activate SIRT-1 and subsequently PGC-1α [19]. Doses of 25 μM RSV have been shown to activate PGC-1α in mouse myoblasts, a process mediated via the metabolic sensor AMP-activated kinase (AMPK) and subsequent SIRT-1 activation [20]. In primary human coronary arterial endothelial cells, 10 μM RSV for 48 h significantly increased mitochondrial mass and mtDNA content, through SIRT-1-mediated induction of the mitogenetic factors PGC-1α, TFAM and nuclear respiratory factor-1 [21]. In mouse myoblasts and embryonic fibroblasts, 20 μM RSV for 48 h stimulated mitochondrial fusion through increased MFN2 expression, resulting in a highly branched mitochondrial network [22]. In vivo, a significantly increased mitochondrial biogenesis was detected in RSV-fed mice [23], an effect comparable to the one achieved by PGC-1α overexpression [24]. In addition, RSV treatment of cultured skin fibroblasts obtained from healthy individuals was shown to stimulate the expression of mitochondrial transcription factor (TFAM) [25]. TFAM is a key factor for mtDNA replication and repair and thus crucial for mitochondrial proliferation.

## 3. The Regulatory Effect of Resveratrol on OXPHOS

In addition to the higher OXPHOS levels that can be achieved by increasing the mitochondrial load, RSV may also interact directly with the OXPHOS chain. Physical association between RSV and OXPHOS complex I has been documented. RSV enters the nucleotide-binding pocket of the NADH dehydrogenase module, competing with NAD+ fixation [26]. Interactions, however, seem to display a complex effect upon OXPHOS activities. NADH oxidation increases with low doses up to 5 μM RSV, while high doses of 50 μM inhibit complex I activity. In vivo, 40 mg/kg/day of RSV significantly increased complex I activity in young mice but not in mice over 22 months old [27]. It has been reported that 100 μM RSV inhibited complex III activity, by competing for binding with co-enzyme Q [28]. Treatment with 20 μM RSV for 48 h increased basal and carbonyl cyanide p-triflouromethoxyphenyl-hydrazone (FCCP) uncoupled respiration rates in mouse myoblasts [22], pointing to a positive effect on complex I to IV activity. Low concentrations (pico to nanoM) activate complex V [29], while doses in the microM range inhibit ATP synthesis [30].

## 4. The Protective Effect of Resveratrol on Oxidative Cell Stress

In the mitochondria, ATP and electrons are produced from NADH and succinate, reducing O_2_ to H_2_O. As a consequence of electron leakage during mitochondrial respiration, O_2_^−^ can be generated through univalent reduction of molecular oxygen. Reactive oxygen species (ROS), which are short-lived molecules with unpaired electrons, are generated. Although low concentrations of ROS serve as second messengers regulating cellular processes, higher concentrations cause oxidative damage. To prevent these harmful effects, aerobic organisms have put a complex array of defensive systems into place, comprising scavenging enzymes and antioxidant agents. In response to oxidative damage, the mitochondrial stress response protein sirtuin-3 (SIRT-3) induces forkhead box O3 (FoxO3a) translocation toward the nucleus, activating antioxidant defense mechanisms through the upregulation of PGC-1α and superoxide dismutase 2 (SOD2). SIRT-3 can directly activate SOD2 via protein/lysine deacetylation [31]. Antioxidant defenses tend to decrease with age, and RSV supplementation has been shown to compensate the SOD2 reduction in mice aged over 22 months [27]. RSV has been reported to trigger cellular anti-oxidant defense mechanisms in aging endothelial cells [32]. In cultured primary human coronary arterial endothelial cells, RSV concentration-dependent increases of the transcriptional activity of nuclear factor-E2-related factor-2 (Nrf2) have been observed [33]. Nrf2 regulates the expression of numerous ROS detoxifying genes. These defense mechanisms also salvage mitochondria from injury caused by hypoxic ischemia, as recently reviewed in [34]. One established experimental model for studying oxidative stress consists of Cd exposure. Using this model, further evidence has been gathered that RSV is capable of restoring ROS homeostasis within cells. In mouse renal tubular epithelial cells, pre-treatment for 2 h with 5 μM RSV protected against Cd-induced ROS elevation and OXPHOS dysfunction. Knockdown experiments with SIRT-3 siRNA have been observed to abolish the protective effect, illustrating the key role such a factor plays [35]. SIRT-3 is known to bind and deacetylate several metabolic and respiratory enzymes that regulate mitochondrial ROS production. [36]. In vivo experiments showed that RSV also protected against Cd-induced perturbations of spermatogenesis and histology in mice [37].

## 5. The Anti-Apoptotic Effect of Resveratrol

Apoptosis or programmed cell death is a highly regulated process that is detrimental to the individual cell, yet possesses crucial constructive potential for tissue development and homeostasis [38]. Mitochondria have a leading role in triggering this process. The association of various regulatory factors including proteins from the B-cell lymphoma 2 (Bcl-2) family, steers, together with the outer mitochondrial membrane, an apoptotic signal cascade transduction either by inducing or by preventing membrane permeabilization. Signaling cell death starts with mitochondrial transmembrane potential depolarization and the release of mitochondrial cytochrome c into the cytosol, which activates cellular proteases (caspases) and leads to subsequent cell apoptosis [39]. Mitochondrial disease has been linked to increased apoptosis. Skeletal muscle tissues of patients with single mtDNA deletions and tRNA point mutations display prominent histologic apoptotic features. Interestingly, the tRNA mutation load matches the degree of apotosis as well as the severity of phenotype [40], identifying apoptosis as a plausible contributing pathogenic mechanism of tissue damage in mitochondrial disorders. Polyphenols have been shown to protect the mitochondrial membrane. RSV pre-incubation prevents hen egg white lysozyme-induced permeabilization in purified mitochondria [41]. An anti-apoptotic effect of RSV has been shown in mouse renal tubular epithelial cells, where pre-treatment with 5 μM RSV reduced extracellular signal-regulated kinases1/2 phosphorylation and prevented Cd-induced activation of caspase-3 activity and Bcl-2-associated X protein (Bax) expression [35].

## 6. Resveratrol Supplementation as a Treatment for Mitochondrial Disorders

**Figure 1:**
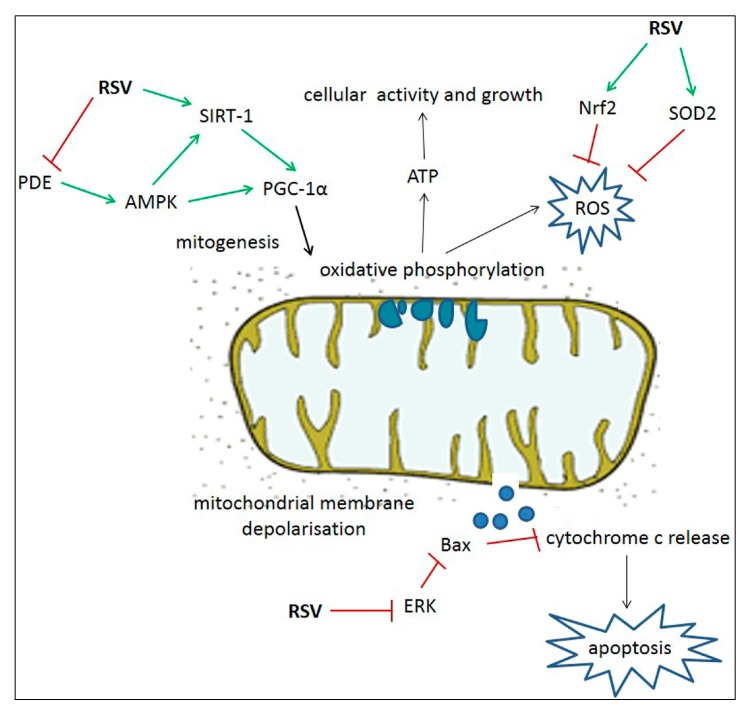
Summary of proposed beneficial effects of resveratrol on mitochondrial respiration and cell survival. Abbreviations: AMP-activated protein kinase (AMPK); B-cell lymphoma 2-associated X protein (Bax); extracellular signal-regulated kinase (ERK); nuclear factor-E2-regulated factor-2 (Nrf2); phosphodiesterase (PDE); peroxisome proliferator-activated receptor-γ coactivator α (PGC-1α); resveratrol (RSV); reactive oxygen species (ROS); sirtuin 1 (SIRT-1); super oxide dismutase 2 (SOD2).

RSV could have its place in a combined therapeutic strategy for boosting OXPHOS in deficient patients, by both stimulating mitochondrial proliferation and reducing cytotoxic ROS by-products and apoptotic cell signaling (Figure 1).

We present here a summary of the results of experiments using primary cultured skin fibroblasts from patients with homoplasmic mtDNA and homozygous or heterozygous nuclear DNA mutations in Table 1.

Only one report is available that assays the effect of RSV on cells with mtDNA defects. In human skin fibroblasts from patients with homoplasmic MT-TL1, MT-TK, and MT-ATP6 gene mutations, 0.01 μM RSV administered for 4 days increased basal oxygen consumption rates and ATP production [44]. A study with cultured skin fibroblasts from patients with OXPHOS complex I and complex IV deficiency showed stimulated mitochondrial biogenesis, as visualized by increased mitotracker green staining intensity and TFAM protein expression, and increased OXPHOS protein levels, which resulted in the correction of the deficiency (measured through oxygen uptake rates) in 6 out of 16 patient cell lines [25]. In cells from patients with complex I deficiency that improved with RSV, treatment resulted in normalized lactate/pyruvate cell supernatant ratios, pointing to restored cellular NADH/NAD ratios. OXPHOS-deficient patients often present with severe lactic acidosis [46]. Another study investigated fibroblasts from 13 patients with nuclear defects causing OXPHOS complex I deficiency, and showed a benefit in administering 75 μM RSV for 48 h in eight patient cell lines. In five cell lines, ROS levels were found to be increased as compared to cells from healthy individuals. RSV treatment significantly reduced ROS levels in four of these patient cell lines, an effect that was attributed to increased SOD2 protein levels established through SIRT-3 activation [43]. Our own study assayed the effect of RSV in cultured skin fibroblasts obtained from patients with either complex II or isolated complex IV deficiency. Although 48 h treatment with 100 μM resveratrol was able to significantly increase complex II and complex IV activities in control cells, activity of the deficient complex was significantly increased in only one of the complex II deficient patients and in none of the complex IV patients [45]. In contrast to the mostly encouraging studies above, treatment of cultured skin fibroblasts from five patients with complex I and/or complex IV deficiency with 25 μM RSV for 72 h was reported to have no or even negative effects upon mitochondrial proliferation, ATP production, and ROS generation [44].

The above-mentioned studies reveal that responses to RSV are not uniform but highly cell-line-dependent. Evidence points toward the magnitude of the OXPHOS defect as an important determinant of beneficial effects that can be registered in cultured fibroblasts. The cohort with varying degrees of OXPHOS deficiency reported by Lopes Costa et al. exhibited significantly increased complex I activities in five cell lines, normalizing values in two of those, while complex IV activity increased significantly only in a cell line carrying a COX10 defect. Interestingly, mean residual complex I and complex IV activities were all above 40% of normal controls in the responsive and below 40% in the non-responsive cell lines [25]. Mathieu et al. reported that the complex I deficient cell lines, which improved in response to RSV, all had residual complex activities between 56% and 28% of normal fibroblasts [43]. Our own study described the significant RSV-induced increase of complex II activity in a patient with residual activities above 20% of normal values, while the two complex II deficient cell lines exhibiting residual activities below 20% of normal did not respond [45].

In summary, the studies published so far indicate a defect-dependent response in cultured skin fibroblasts, displaying an approximately 50/50 chance of deficient cells benefitting from RSV treatment. Data indicate that cells might still require substantial remaining residual OXPHOS activities to generate a response.

## 7. Conclusions

RSV displays multiple interactions with mitochondrial activity and unmistakably possesses mitogenetic, anti-oxidant, and anti-apoptotic effects. The compound is a small lipophilic molecule that can cross cell barriers and penetrate cell organelles with ease. Therapeutic success appears, however, to be variable and dependent on many factors, which include the severity of the underlying defect and the administered dose. Importantly, a threshold of residual OXPHOS activity seems necessary for enabling RSV to generate a response. Therefore, the life-saving potential of RSV in mitochondrial disease seems extremely limited. RSV could, however, be beneficial to individual patients when used as a supportive therapeutic supplement and as part of a multi-component therapy. In support, supplements of 500 mg/day RSV have been shown to enhance exercise-induced improvement of muscle capacities in subjects 65 years or older [47].

## Figures and Tables

**Table 1:** In vitro results of resveratrol treatment outcome in cultured skin fibroblasts from patients with oxidative phosphorylation defects.

Mutation	Deficiency	Regimen	Outcome	Reference
m.3243A>G TL1	not reported	0.01 μM 24 h	basal OCR and ATP production significantly ↑	[42]
m.8344A>G TK	not reported	0.01 μM 24 h	basal OCR and ATP production significantly ↑	[42]
m.8993T>G ATP6	not reported	0.01 μM 24 h	basal OCR and ATP production significantly ↑	[42]
c.611A>G NDUFV1	complex I	75 μM 48 h 72 h	OCR and complex I activity significantly ↑	[25]
c.640G>A NDUFV1	complex I	75 μM 48 h 72 h	normalized complex I activity	[25]
c.1294G>C NDUFV1	complex I	75 μM 48 h 72 h	OCR and complex I activity significantly ↑	[25]
c.1129G>A NDUFV1	complex I	75 μM 48 h 72 h	no change	[25]
c.1142A>G c.11G>A NDUFV1	complex I	75 μM 48 h	no change	[43]
c.1156C>T NDUFV1	complex I	75 μM 48 h	basal OCR, ATP production and complex I activity significantly ↑	[43]
c.1157G>A NDUFV1	complex I	75 μM 48 h 72 h	no change	[25]
c.54X>A c.207dup NDUFV2	complex I	75 μM 48 h	basal OCR significantly ↑	[43]
c.120+5_120+8delGTTA NDUFV2	complex I	75 μM 48 h 72 h	basal OCR, ATP production and complex I activity significantly ↑	[43]
gene deletion NDUFS1	complex I	75 μM 48 h 72 h	no change	[25]
c.683T>C c.755A>C NDUFS1	complex I	75 μM 48 h	basal OCR significantly ↑	[43]
c.721C>T NDUFS1	complex I	75 μM 48 h 72 h	no change	[25]
c.1139A>T c.63+6T>G NDUFS1	complex I	75 μM 48 h	no change	[43]
c.875T>C c.1328T>A NDUFS2	complex I	75 μM 48 h	basal OCR and complex I activity significantly ↑	[43]
c.875T>C c.353G>A NDUFS2	complex I	75 μM 48 h	no change	[43]
c.1237T>C NDUFS2	complex I	75 μM 48 h	no change	[43]
c.434C>T NDUFS3	complex I	75 μM 48 h 72 h	no change	[25]
c.291delG NDUFS4	complex I	75 μM 48 h	basal OCR, ATP production and complex I activity significantly ↑	[43]
c.67del NDUFS6	complex I	75 μM 48 h	basal OCR significantly ↑	[43]
c.17-1167C>G NDUFS7	complex I	75 μM 48 h	no change	[43]
c.434G>A NDUFS7	complex I	75 μM 48 h	basal OCR and complex I activity significantly ↑	[43]
unknown	complex I	75 μM 48 h 72 h	normalized complex I activity	[25]
unknown	complex I	25 μM 72 h	no change	[44]
c.296A>G C8orf38	complex I	75 μM 48 h 72 h	no change	[25]
c.997C>T EFTs	complex I & IV	25 μM 72 h	ATP production and mitochondrial content significantly ↑	[44]
c.509G>A MRPS22	complex I & IV	25 μM 72 h	ATP production significantly ↓ ROS significantly ↑	[44]
unknown	complex II	100 μM 48 h	no change	[45]
c.622G>T NFU1	complex II	100 μM 48 h	no change	[45]
c.1663G>A Fp	complex II	100 μM 48 h	complex II and CS activity significantly ↑	[45]
unknown	complex IV	100 μM 48 h	no change	[45]
unknown	complex IV	100 μM 48 h	complex II and CS activity significantly ↑	[45]
unknown	complex IV	100 μM 48 h	complex II and CS activity significantly ↑	[45]
c.312_321del insAT SURF-1	complex IV	100 μM 48 h	complex IV and CS activity significantly ↑	[45]
c.312_321del insAT SURF-1	complex IV	100 μM 48 h	no change	[45]
c.312_321del insAT SURF-1	complex IV	100 μM 48 h	CS activity significantly ↑	[45]
c.312_321del10insAT SURF-1	complex IV	75 μM 48 h 72 h	no change	[25]
c.539G>A SURF-1	complex IV	75 μM 48 h 72 h	no change	[25]
c.845_846delCT SURF-1	complex IV	100 μM 48 h	no change	[45]
c.612C>A	complex IV	75 μM 48 h 72 h	no change	[25]
c.36142203C>T COX6B1	complex IV	25 μM 72 h	mitochondrial content and ROS significantly ↑	[44]
c.612C>A COX10	complex IV	75 μM 48 h 72 h	complex IV activity significantly ↑	[25]
unknown	Complex IV	25 μM 72 h	no change	[44]
unknown	complex IV	100 μM 48 h	complex II and CS activity significantly↑	[45]

Increases are indicated with ↑, decreases with ↓. Abbreviations: citrate synthase (CS); cytochrome c-oxidase (COX); elongation factor for mitochondrial translation (EFT); mitochondrial ribosomal protein (MRP); NADH:ubiquinone oxidoreductase core subunit (NDUF); iron-sulfur cluster scaffold (NFU); oxygen consumption rate (OCR); oxidative phosphorylation (OXPHOS); surfeit locus protein-1 (SURF-1); tRNA lysine (TK); tRNA leucine (TL).
